# Supplementary material for: Improving Engagement in Antenatal Health Behavior Programs—Experiences of Women Who Did Not Attend a Healthy Lifestyle Telephone Coaching Program
Source: Nutrients. 2023 Apr 12;15(8):1860. doi: 10.3390/nu15081860 (PMC10146126; doi:10.3390/nu15081860)
Supplement: Supplementary file 1 [file nutrients-15-01860-s001.zip › Interview schedule S1.pdf]

Semi-structured Interview Guide and Prompts

Enhancing engagement and early access to the Living Well during Pregnancy Program: what do women need?

Introduction

Hi _____, thank you for taking the time to talk to me today. As part of our ongoing service improvement our team is looking at how we can better engage and support women with nutrition, exercise, and weight gain during their pregnancies. We encourage you to be open and honest throughout the discussion to help us ensure our service provides the support women need.

We are interested in your thoughts about nutrition, exercise, and weight gain during pregnancy and the support you received, or wanted to receive, from the hospital.

If attended 1-2 appointments:
We would also like to hear about your experience with the Living Well during Pregnancy phone coaching service and if there is anything we can do to improve the delivery of this service for women during their pregnancy.

The interview should take no more than 30 minutes. If at any time you would like to withdraw from the project or the interview to stop, please let me know. You are under no obligation to continue with the interview if you are feeling uncomfortable.

To ensure all the information you provide is understood and remembered, and so I can focus on you and our discussion, we would like to record the interview. I have a program that will record our conversation. Our conversation and the recording will remain confidential, with none of your personal information recorded or identified during our analysis.

Are you happy to consent to this interview being recorded?

Do you have any questions before we begin?

Theme: Experiences with information and help on nutrition, exercise, and weight gain during pregnancy

Question	Probe
To start, did the health professionals you saw throughout your pregnancy, such as your GP, midwife, or obstetrician, speak to you about food, exercise, or weight gain during your pregnancy?	What did they say or recommend?<br><br>How did you feel about these recommendations?<br><br>Did they give you any information or refer you to other services?
Outside of the health professionals you saw throughout your pregnancy, was there anywhere else you looked for information or help with nutrition, exercise, or weight gain?	Who provided the information/support?<br><br>Who/what made you feel the most supported?

Was there any other type of information or help that you would have liked to receive on nutrition, exercise, or weight gain during pregnancy?<br><br>Is there any other information or help that other mums might want on nutrition, exercise, or weight gain during pregnancy?	Where/what format (digital, books etc)/ who did you look to for the information?
**Theme: Experiences with Living Well during Pregnancy**	
Question	Probe
During your latest pregnancy you were referred into the Living Well during Pregnancy program, could you tell me about how you first heard about the program?<br><br>What were your initial thoughts about the program?<br><br>How many, if any, of the sessions did you attend?	What information was provided?<br><br>Can you tell me about that experience/why that is?
**If attended at least one session:**<br><br>For the session/s you attended, was their anything you took away from them?<br><br>From your experience, is there anything we can change with the program to provide the information and help mothers want with nutrition, exercise, or weight gain during pregnancy?	**If did not attend any sessions:**<br><br>Is there anything we could improve or change in the program to help other mum's in a similar situation to you receive information on nutrition, exercise, or weight gain during pregnancy?
**Theme: Ideal support services**	
Question	Probe
What do you think is the best way for dietitians or health coaches to provide information and help to women on nutrition, exercise, and weight gain during pregnancy?<br><br>Have you had any experience with digital health support services? E.g. text messages, emails, videos, support groups	What style of support was most helpful to you?

If money was no object, what would be the ultimate service that we could provide women to help them eat well, exercise, and gain weight within the guidelines during pregnancy?	
Is there anything else you'd like to add?	
Thank you again for taking the time to talk with me today. After our call today I will email you a $20 eGift card as an appreciation for your time to the email you provided during registration. If you could please reply to my email to let me know you have received the gift card that would be appreciated.<br><br>If you had any questions about the progress of this project, please feel free to send me an email.<br><br>Thank you again!	
