# Supplementary material for: Improving Engagement in Antenatal Health Behavior Programs—Experiences of Women Who Did Not Attend a Healthy Lifestyle Telephone Coaching Program
Source: Nutrients. 2023 Apr 12;15(8):1860. doi: 10.3390/nu15081860 (PMC10146126; doi:10.3390/nu15081860)
Supplement: Supplementary file 1 [file nutrients-15-01860-s001.zip › Questionnaire S1.pdf]

ONLINE REGISTRATION

Potential participant arrives at online registration form via entering link

**Page 1**

INTRODUCTION

Enhancing engagement and early access to the Living Well during Pregnancy Program: what do women need?

Thank you for your interest in this project.

This survey will take approximately 10-15 minutes to complete, including the time to read the Participant Information and Consent Form which will appear on the next page.

NEXT >

**Page 2**

PARTICIPANT INFORMATION AND CONSENT

<Insert PICF and link to download PDF copy>

NEXT >

**Page 3**

PARTICIPANT SURVEY

First, we will ask you some questions about yourself. If you decide to withdraw from this project before submitting the final registration information and consent to be contacted, we will not keep your data.

By clicking ‘next’, you agree to participate in the participant survey which will take no more than 5 minutes.

NEXT >

Page 4

ABOUT YOU

1) **How old are you (in years)?**

2) **What is your highest level of education?**

- o Less than Year 12 or equivalent
- o Year 12 or equivalent
- o Vocational Qualification
- o Associate or undergraduate diploma
- o Bachelor degree (including honours)
- o Postgraduate diploma
- o Postgraduate degree

3) **What is your approximate average household income?**

- o $ 0 - $49,999
- o $50,000 - $99,999
- o $100,000 - $149,999
- o $150,000 - $199,999
- o $200,000 and up

4) **Where were you born?**

- o Australia
- o Other English speaking country
- o Other non-English speaking country

5) **What is the primary language spoken at home? (text box)**

6) **How many times have you been pregnant? (numbered drop-down list)**

7) **How many children do you have? (numbered drop-down list)**

NEXT >

**Page 5**

TELEPHONE INTERVIEW REGISTRATION

By completing the following details, you consent to be contacted for a telephone interview. It also means you have read the Participant Information and Consent Form and filled in the survey. It does not mean you need to complete the interview. You can withdraw from the project at any stage.

First name:

Best contact number:

Email address (for alternative contact):

Please list any days (between Monday and Friday) or times that you will not be able to answer the phone:

Please note, that the call you receive from us may show as ‘No Caller ID’. If we contact you at an inconvenient time, we will arrange a better time to speak with you. You can expect the telephone interview to take around 20 to 30 minutes.

NEXT >

Page 6

Thank you for completing the survey and registering your interest in participating in a telephone interview. We will be in touch soon via the phone number you provided. If you have any questions or concerns or would like to withdraw your information or interest, please contact the Project Lead (see below).

Project Lead: Jessica Fry, Dietitian

Jessica.Fry@health.qld.gov.au

(07) 3646 8268
