# Supplementary material for: Improving Engagement in Antenatal Health Behavior Programs—Experiences of Women Who Did Not Attend a Healthy Lifestyle Telephone Coaching Program
Source: Nutrients. 2023 Apr 12;15(8):1860. doi: 10.3390/nu15081860 (PMC10146126; doi:10.3390/nu15081860)
Supplement: Supplementary file 1 [file nutrients-15-01860-s001.zip › Table S1.pdf]

Table S1 – Non-responders characteristics (n=148)

**Variable**	**Median (range)/Number (%)**
Age (years)	33 (21-46)
Gravida	
1	54 (36)
2	41 (28)
3	23 (16)
4	17 (11)
5	5 (3)
6	5 (3)
7	1 (1)
9	1 (1)
11	1 (1)
Parity	
0	85 (57)
1	39 (26)
2	13 (9)
3	9 (6)
5	1 (1)
7	1 (1)
Program Appointments Attended	
0	65 (44)
1	58 (39)
2	25 (17)
